# Rapid bioerosion in a tropical upwelling coral reef

**DOI:** 10.1371/journal.pone.0202887

**Published:** 2018-09-12

**Authors:** André Wizemann, Sri D. Nandini, Ines Stuhldreier, Celeste Sánchez-Noguera, Max Wisshak, Hildegard Westphal, Tim Rixen, Christian Wild, Claire E. Reymond

**Affiliations:** 1 Leibniz Centre for Tropical Marine Research, ZMT, Bremen, Germany; 2 University of Bremen, MARUM, Center for Marine Environmental Sciences, Bremen, Germany; 3 University of Bremen, Faculty of Geosciences, Bremen, Germany; 4 Centro de Investigación en Ciencias del Mar y Limnología, CIMAR, Universidad de Costa Rica, San Pedro de Montes de Oca, San José, Costa Rica; 5 Senckenberg am Meer, Wilhelmshaven, Germany; 6 University of Bremen, Faculty of Biology & Chemistry, Marine Ecology Department, Bremen, Germany; Havforskningsinstituttet, NORWAY

## Abstract

Coral reefs persist in an accretion-erosion balance, which is critical for understanding the natural variability of sediment production, reef accretion, and their effects on the carbonate budget. Bioerosion (i.e. biodegradation of substrate) and encrustation (i.e. calcified overgrowth on substrate) influence the carbonate budget and the ecological functions of coral reefs, by substrate formation/consolidation/erosion, food availability and nutrient cycling. This study investigates settlement succession and carbonate budget change by bioeroding and encrusting calcifying organisms on experimentally deployed coral substrates (skeletal fragments of *Stylophora pistillata* branches). The substrates were deployed in a marginal coral reef located in the Gulf of Papagayo (Costa Rica, Eastern Tropical Pacific) for four months during the northern winter upwelling period (December 2013 to March 2014), and consecutively sampled after each month. Due to the upwelling environmental conditions within the Eastern Tropical Pacific, this region serves as a natural laboratory to study ecological processes such as bioerosion, which may reflect climate change scenarios. Time-series analyses showed a rapid settlement of bioeroders, particularly of lithophagine bivalves of the genus *Lithophaga*/*Leiosolenus* (Dillwyn, 1817), within the first two months of exposure. The observed enhanced calcium carbonate loss of coral substrate (>30%) may influence seawater carbon chemistry. This is evident by measurements of an elevated seawater pH (>8.2) and aragonite saturation state (Ω_arag_ >3) at Matapalo Reef during the upwelling period, when compared to a previous upwelling event observed at a nearby site in distance to a coral reef (Marina Papagayo). Due to the resulting local carbonate buffer effect of the seawater, an influx of atmospheric CO_2_ into reef waters was observed. Substrates showed no secondary cements in thin-section analyses, despite constant seawater carbonate oversaturation (Ω_arag_ >2.8) during the field experiment. Micro Computerized Tomography (μCT) scans and microcast-embeddings of the substrates revealed that the carbonate loss was primarily due to internal macrobioerosion and an increase in microbioerosion. This study emphasizes the interconnected effects of upwelling and carbonate bioerosion on the reef carbonate budget and the ecological turnovers of carbonate producers in tropical coral reefs under environmental change.

## Introduction

Tropical coral reefs are among the most productive biogenic calcium carbonate (CaCO_3_) producing ecosystems in the world. At the same time the biogenic skeletal CaCO_3_ is degraded by means of bioerosion [1], rendering this process an integral component of the CaCO_3_ budget. CaCO_3_ bioerosion is a dynamic process pertaining to complex ecological impacts within coral reefs [2]. The intensity and pace of bioerosion influences the cycling of biogenic CaCO_3_ and supports the formation of sediment in large buildups such as carbonate platforms and reef structures [3–5]. From the reef ecosystem or colony scale, bioerosion, by way of endolithic (i.e. inside hard substrate) micro- and macrobioerosion, as well as epilithic (i.e. on hard substrate) attachment etching and grazing activity, effects the physical resistance of coral reef framework to extrinsic erosion such as storm surges, thereby further promoting sediment production [6]. However, calcifying bioeroding and encrusting species also bind and cement loose sediments (i.e. form calcareous overgrowth), and create new habitats with consolidated substrate [7,8]. In most tropical oligotrophic settings colonization of coral skeletons by bioeroders and encrusters typically occurs within days and is considered to develop a mature community within several months to years [9]. In marginal tropical reef systems colonization and development of a community may be even more rapid and intense. Many marginal reefs are exposed to pronounced environmental changes such as meridional migration of the circulation systems in the ocean and the atmosphere [10]. Upwelling systems can influence such reef ecosystems, temporarily favoring organotrophic composed carbonate communities [11–14]. Typically, the ensuing marginal reef settings are non-framework or low-relief coral communities [15]. Marginal reefs present an excellent opportunity to investigate carbonate dynamics over time, as transitions in the reef community may occur on a regular base [16]. This is pertinent to study as reef bioerosion processes are expected to accelerate under future ocean acidification [17–19] and eutrophication scenarios [20].

The aim of this study is to investigate how upwelling influences bioerosion patterns and the CaCO_3_ budget of bioerosion on substrates in a marginal reef setting located in the Gulf of Papagayo, Costa Rica, Eastern Tropical Pacific (ETP). Therefore, skeletal coral substrates were placed onto the benthic cover in a local coral reef during the upwelling season from December 2013 to March 2014. Monthly recovery of substrates enabled the documentation of the bioeroder and encruster succession at a high temporal resolution. For analysis of macro- and microbioerosion patterns, Micro Computerized Tomography (μCT), thin-sections and cast-embeddings were used together with Scanning Electron Microscopy (SEM). Concomitant measurements of the seawater parameters such as nutrients, temperature, pH, dissolved inorganic carbon (DIC) and total alkalinity (A_T_) with calculations of the bioerosion CaCO_3_ budget of substrates (net CaCO_3_ weight change) allowed further discussion on the correlation of the bioerosive activity to the influence of the ambient seawater properties. Finally, a conceptual environmental model illustrates how bioerosion processes take part in the functioning of marginal reef ecosystems in the ETP.

## Materials and methods

### Environmental setting and study site

**Fig 1:**
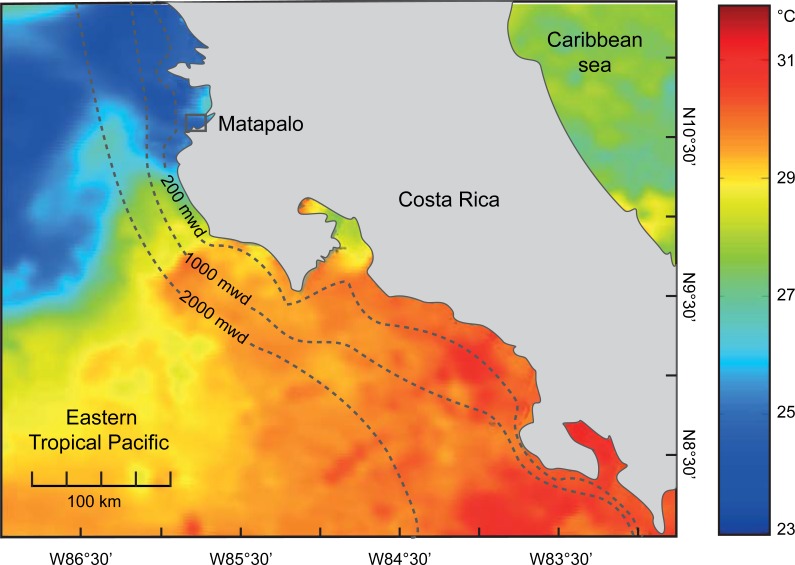
Map of the eastern tropical pacific coast of costa rica with the location of the study site, Matapalo Reef (10°32’21”N, 85°45’59”W), in the Gulf of Papagayo (small inset). Mean sea surface temperatures (SST) on the right side indicate the oceanic hydrothermal setting during the major upwelling period (17 February 2014). The SST data were derived from daily global maps with a grid map resolution of 1 km (GHRSST, Level 4, G1SST) produced by the JPL Regional Ocean Modeling System group available from http://ourocean.jpl.nasa.gov/SST/. The data was visualized with the Ocean Data View software.

The ETP is one of the most productive tropical marine regions due to upwelling of macronutrient-rich subsurface waters into the euphotic zone [21,22]. All along the ETP, continental shelf coral reef ecosystems have developed within the periphery of the optimal environmental conditions for coral growth (in respect to thermal range and turbidity). One of the larger tropical coral reefs off the Pacific coast of Costa Rica is located in the semi-sheltered Bay of Matapalo, which is part of the Gulf of Papagayo (Fig 1) [23,24].

During the dry season (December-April; northern winter), the Gulf of Papagayo is exposed to upwelling when the Papagayo jet, a trade wind from the mainland, intensifies (Fig 1) [25–27]. During this period, wind-driven upwelling and the seasonal extension of the Costa Rica Dome brings cool (22–26°C), low pH (<8), and nutrient-rich subsurface water into the Gulf of Papagayo [28,29]. Consequently these conditions allow the formation of extensive but poorly developed reefs [24]. In these reefs, bioerosion is an integral part of the reef framework and carbonate sediment production [30,31]. Sediments at the reef site were comprised of dead coral branches of the genus *Pocillopora* alternating with patches of fine carbonate sand (S2 Fig). Such fields of coral rubble form typical substrate of many reefs within the ETP [32,33].

### Pre-experimental preparations

Similar to the sedimentary substrate at the study site (i.e. coral rubble of *Pocillopora* branches; S2 Fig), skeletal framework of a dead *Stylophora pistillata* grown in the marine experimental facility at the Leibniz Centre for Tropical Marine Research (ZMT) in Germany was used in the field experiment (CITES permit number 10314/IV/SATS-LN/2009). Despite being non-native in the ETP, *S*. *pistillata* is a branching species with calices of comparable size (within a range of ~1.0 to 1.5 mm; e.g. [34]). This coral colony was cut into small cylindrical blocks of approximately 1 cm in diameter and 3 cm in length. To remove any soluble components and organic tissue, the coral substrates were cleaned for 48 h with hydrogen peroxide (H_2_O_2_ 30%). This was done to avoid abnormal causes for an attraction of bioerosive/encrusting settlers (e.g. molecular/organic sensorial attraction). Subsequently, the cleaned substrates were weighed (Mettler Toledo, AT 21 Comperator; accuracy >0.1 mg) before being deployed in the reef.

### Experimental setup

Exposure experiments were conducted during the northern winter upwelling period from December 2013 to April 2014. For this purpose, a total of 16 *S*. *pistillata* substrates were fixed within custom made plastic frames with angler line, whereby a hole was drilled pre-experimentally in the middle part of each substrate (S1 Fig). The frames were placed at Matapalo Reef ~5 m below sea level (bsl) and suspended approximately 0.5 m above the seafloor. To allow undisturbed settlement the coral substrates were uncaged. To identify settling succession and CaCO_3_ erosion rates, four replicate coral substrates were retrieved consecutively after one, two, three, and four months of exposure, respectively. However, over the exposure period four of the coral substrates were lost due to external forces (e.g. currents, fish bites, crumbling) resulting in a reduced number of replicates for some of the months. Originally, substrates were deployed in a higher temporal replication at the described study site and also at Bahía Santa Elena (10°56’526”N, 85°48’838”W), located north of Matapalo Reef. Due to major loss of substrates, this study has to focus on the results from Matapalo Reef during the upwelling period. S10 Fig exemplarily presents one substrate deployed at Bahía Santa Elena on December 11^th^ 2013. The sample was recovered on February 13^th^ 2014 after two months of exposure. Other substrates deployed at Bahía Santa Elena were lost after the second month.

### Water parameter measurements

#### Nutrient concentration, physico-chemical seawater parameters

Seawater nutrient concentration and physico-chemical parameters, such as seawater temperature and salinity, were measured by Stuhldreier et al. [23] directly above the reef substrate on a weekly basis. Total scale pH (pH_Manta_) was measured between December 2013 and April 2014 by deploying a Manta 2 Water Quality Multiprobe (Eureka Environmental Engineering) 0.5 m above the reef substrate. Stuhldreier et al. [23] provided further details regarding data processing. Since the pH_Manta_ measurements did not meet the accuracy requested in Dickson et al. [35], discrete water samples were collected during daytime next to the Manta multiprobe at a water depth of ~6 m. Occasionally, additional surface water samples were collected at a depth of 0.5 m. The results obtained from the surface and bottom water samples were averaged and are presented in Table 1. Total alkalinity (A_T_) and total dissolved inorganic carbon (DIC) were determined with a titration unit VINDTA 3C (Marianda, Kiel, Germany), which includes a UIC CO_2_ coulometer detector (UIC Inc., Joliet, USA). The VINDTA 3C was calibrated using the Dickson Certified Reference Material (Batch 127) [36]. Sánchez-Noguera et al. [37] describe the method in further detail. This method meets the requested standard [35] and the program CO_2_SYS was used to calculate the pH_VINDTA_ (total scale), the *p*CO_2_ and aragonite saturation state (Ω_arag_). For the calculations, the daily mean seawater temperature and salinity obtained from the Manta multiprobe were used, except on February 3^rd^ and March 31^st^ 2014. At these two days the Manta multiprobe was not deployed and a WTW sensor was used to measure seawater temperature and salinity [37].

**Table 1:** Monitored and calculated (pH_VINDTA_, *f*CO_2_, Ω_arag_) seawater parameters for carbon chemistry at the study site of Matapalo Reef, Costa Rica. See also S2 Table and Fig 2A for comparison of pH_Manta_ and pH_VINDTA_.

Date (d/m/y)	Time	Depth (m)	A_T_ (μmol/kg)	DIC (μmol/kg)	SST (°C)	SSS	pH-cal (total scale)	fCO_2_-cal (μatm)	Ω_arag_-cal
02/12/2013	16:10	6.00	2211.18	1971.99	25.63	32.52	7.97	479.48	2.81
09/12/2013	15:30	6.00	2106.38	1805.37	27.87	31.08	8.09	330.82	3.49
16/12/2013	15:30	6.00	2093.72	1822.51	28.20	31.00	8.03	385.15	3.18
23/12/2013	15:30	6.00	2072.18	1785.09	28.16	30.68	8.07	343.27	3.35
30/12/2013	15:30	6.00	2078.75	1783.16	28.53	30.70	8.08	335.76	3.45
06/01/2014	14:30	5.00	2086.62	1789.08	28.51	31.19	8.07	339.57	3.45
20/01/2014	13:13	3.25	2213.52	1890.27	26.41	31.92	8.12	318.66	3.73
21/01/2014	08:30	3.25	2218.88	1918.48	26.41	30.54	8.10	345.66	3.55
23/01/2014	11:23	3.25	2209.45	1917.96	26.64	33.31	8.04	391.36	3.34
24/01/2014	13:00	3.25	2224.72	1938.93	25.98	32.02	8.06	381.87	3.33
25/01/2014	11:45	3.25	2207.52	1903.20	26.80	30.50	8.10	340.08	3.59
26/01/2014	12:15	3.25	2169.64	1864.63	27.46	32.98	8.06	360.27	3.49
27/01/2014	12:30	3.25	2185.60	1874.89	27.19	33.13	8.07	353.02	3.55
28/01/2014	08:50	3.25	2198.65	1915.50	27.00	33.90	8.02	412.88	3.24
03/02/2014	12:30	5.50	2179.02	1889.40	27.00	33.65	8.03	390.48	3.30
31/03/2014	12:28	3.00	2256.14	1924.72	25.40	33.67	8.11	337.41	3.75
17/04/2014	10:02	2.25	2263.01	1956.66	27.95	33.64	8.03	404.54	3.55

### Post-experimental sample treatment and bioerosion CaCO_3_ substrate budget analyses

All coral substrates retrieved were air dried and shipped back to ZMT for further analyses. At ZMT, the coral substrates were digitally photographed and weighed after bleaching with H_2_O_2_ (30%) for 72 h, which removed organic material (S9 Fig). Net erosion rates were calculated from the weight loss of the substrate (normalized to milligrams of CaCO_3_ removed per substrate and day). Additionally, percentages of CaCO_3_ loss rate per substrate, and monthly means were calculated. A one-way ANOVA test using JMP (version 9.0.2) was conducted to statistically assess the change in CaCO_3_ during the four months exposure period. Homogeneity of variance of the means is assumed (F_3,8_ = 4.10) based on the Levene’s test (Prob > F = 0.05) followed by a Tukey HSD means comparison for each month, which distinguished if means were significantly different from each other. However, it is noted that there is a small sample size and therefore a likelihood of a type II error.

### Micro Computerized Tomography (μCT) scanning

Micro Computerized Tomography (μCT) scans were conducted from one control substrate (pre-experiment) and from one substrate of each exposure period (i.e. from each of the monthly recoveries) throughout the field experiment. On top of the substrates a small CaCO_3_ body was mounted with modeling clay to facilitate beam hardening correction during the reconstruction process. Substrates were scanned using a Skyscan^®^ 1772 μCT scanner (located at Kiel University; Department of Geoscience) with a voxel size of 7–8 μm in 0.9 mm rotational steps and 360° rotation. The raw scan data was reconstructed at ZMT Bremen using the software nRecon with 43% beam hardening correction, no data smoothing and maximum ring artifact reduction accuracy. Voxel-based 3D volume models were visualized with the software CTVox and a color map was applied to discriminate morphological changes due to encrustation and bioerosion (S4–S8 Figs; S10 Fig).

### Microbioerosion analyses

Microbioerosion was investigated using Scanning Electron Microscopy (SEM) of cast-embeddings and petrographic thin-sections of the coral substrates. Partially etched (5% HCl solution for approx. 30s) epoxy-resin casts were prepared in a vacuum chamber following the protocol in Wisshak [38], except for the application of an alternative epoxy resin (R & G cast resin “water-clear” UN3082 + 2735). The casts, showing the positive infill of the bioerosion traces were rinsed with purified water, dried, mounted, and sputter-coated with gold for investigation by SEM with the use of the secondary electron detector at 20 keV (Tescan Vega3 XMU).

For the investigation of microbioerosion from thin-sections, longitudinal and latitudinal petrographic thin-sections of the previously μCT scanned coral substrates were prepared. For this, substrates were embedded in epoxy and subsequently sections were polished to a thickness of 45 μm. Thin-sections for SEM analyses were gold-sputtered for 30 s and analyzed using the Back-Scattered Electron detector (BSE) at 10 keV.

For analyses of surface microbioerosion, coral substrates were mounted on SEM stubs with conductible modeling clay (Leit-C plast). The surface of the substrates was then examined using low-vacuum mode and the BSE detector at 20 keV.

## Results

### Physico-chemical seawater parameters

**Fig 2:**
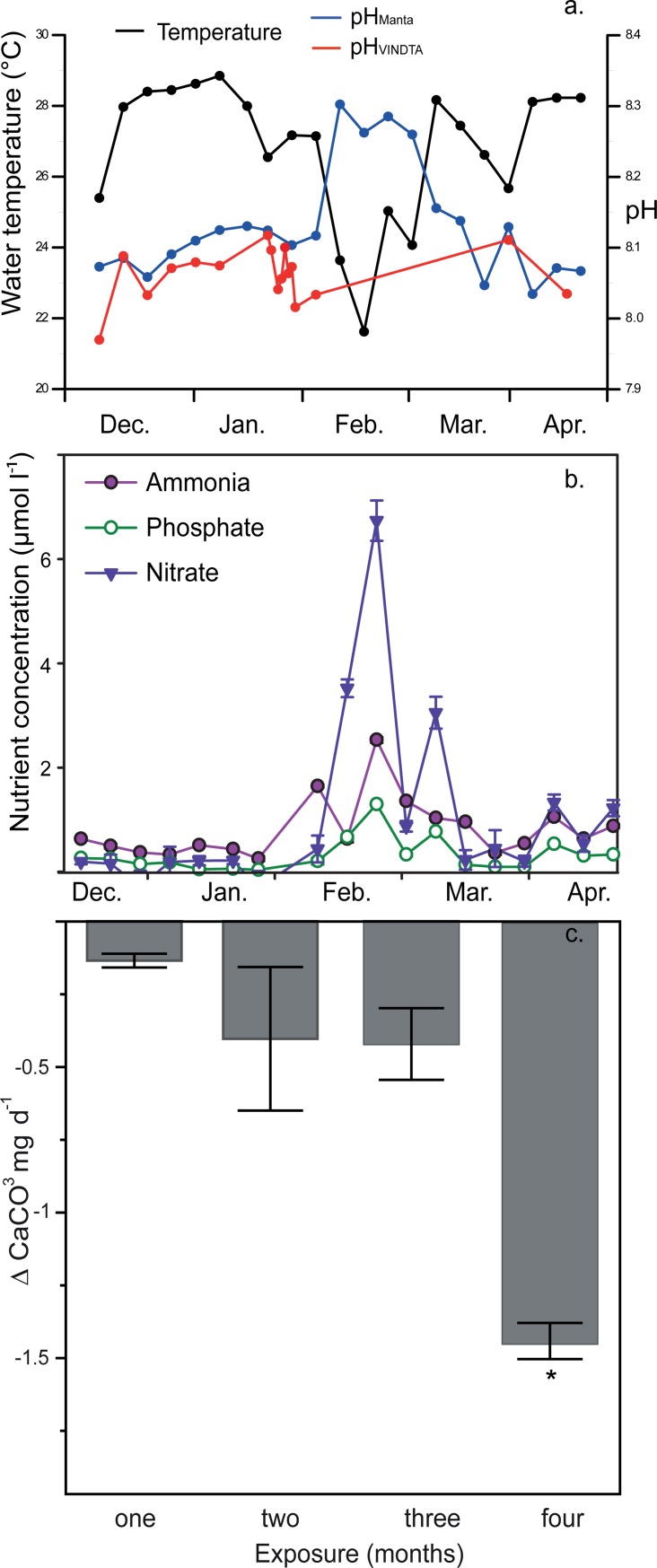
Graphs showing a) daytime means of seawater temperature, pH_Manta_ (total scale) and pH_VINDTA_ (total scale), b) nutrient concentrations of nitrate, ammonia and phosphate, and c) bioerosion CaCO_3_ budget of the experimental coral substrate through time (with standard deviation, black bars). Temperature, pH_Manta_ and nutrient data modified after Stuhldreier et al. [23].

Mean seawater temperature during the first two months (December 2013 to January 2014) was 27.2°C (Fig 2A). In February 2014 seawater temperature dropped down to 21.6°C. This temperature decrease was accompanied by increasing concentrations of dissolved nutrients, indicating a major upwelling event (i.e. cold water intrusions), which lasted for about three to four weeks (Fig 2). In 2009, a similar upwelling event was observed 15 km to the northeast at Marina Papagayo, at a site within ~200 m distance to a coral reef, where mean seawater temperature decreased from about 26.3°C to 23.7°C [39]. Since oxygen-depleted and nutrient-enriched subsurface waters are corrosive [40], seawater pH decreased and *p*CO_2_ increased during this upwelling event in 2009. In contrast, during the upwelling event observed in February 2014 at Matapalo Reef, the pH_Manta_ increased from 8.11 to 8.30 (Fig 2A). Unfortunately, no DIC and A_T_ data were obtained during this pronounced upwelling event in 2014 (Fig 2A, Table 1, S2 Table). Prior to and after the upwelling event, pH_Manta_ corresponded with the pH_VINDTA_ derived from DIC and A_T_ measurements. Thus, it is unlikely that the increase of pH_Manta_ during the upwelling is a measurement error. A pH of up to 8.3 was not measured at Marina Papagayo during 2009, 2012 and 2013 [37,39]. Even if this pH_Manta_ reading is considered as erroneously high, it indicates that the pH did rise during the 2014 upwelling event, and not drop as expected. The pH_VINDTA_ derived from DIC and A_T_ measurements represent daytime values. State of the art pH measurements [35] at Marina Papagayo (pH 7.9–8.05) during the non-upwelling periods in 2012 and 2013, [37] and the upwelling event in 2009 [39] indicated a diurnal pH variability of less than ±0.15. During the non-upwelling periods the pH was generally lower at night and increased from the early morning hours until the late afternoon. During the upwelling season the intrusion of corrosive subsurface water largely masked the diurnal trend [39].

At Matapalo Reef, Ω_arag_ derived from A_T_ and DIC measurement ranged between 2.8 and 3.7 (mean 3.4 ±0.2; Table 1) over the experimental period and mostly exceeded the global means of ~2.9 [41]. The *f*CO_2_ varied between 318.7 and 479.5 μatm with an average of 367.7 ±40.4 μatm (Table 1). During the period of observation the atmospheric CO_2_ concentrations increased from ~394 to ~401 ppm as measured at Mauna Loa in the central Pacific Ocean (NOAA, Earth System Research Laboratory, Global Monitoring Division). This indicates an influx of atmospheric CO_2_ into the seawater surrounding Matapalo Reef. In contrast, during the upwelling event in 2009 at Marina Papagayo [39] seawater *p*CO_2_ exceeded atmospheric CO_2_ and thus CO_2_ was emitted. In addition to upwelling, the intrusion of subsurface water via enhanced wind mixing increased seawater *p*CO_2_ from ~320 μatm to ~600 μatm during the non-upwelling period in 2009 [39], similar to observations in 2012 [37].

### Nutrient concentrations

Mean concentrations of nitrate were 0.09 ± 0.10 μmol/L in the first month, 0.97 ± 0.87 μmol/L in the second month, 2.72 ± 1.47 μmol/L in the third month (upwelling pulse), and 0.63 ± 0.24 μmol/L in the fourth month (Fig 2B). With the onset of upwelling during the third month, nitrate concentrations peaked at 6 μmol/L (Fig 2B). Mean concentrations of ammonia were 0.47 ± 0.05 μmol/L in the first month, 0.74 ± 0.31 μmol/L in the second month, 1.47 ± 0.36 μmol/L in the third month (upwelling pulse), and 0.65 ± 0.01 μmol/L in the fourth month (Fig 2B). Concentrations of ammonia peaked in the third month at ~3 μmol/L corresponding with the onset of upwelling (Fig 2B). Mean concentrations of phosphate were 0.18 ± 0.04 μmol/L in the first month, 0.24 ± 0.15 μmol/L in the second month, 0.63 ± 0.26 μmol/L in the third month (upwelling pulse), and 0.26 ± 0.11 μmol/L in the fourth month (Fig 2B). Concentrations of phosphate peaked at ~1 μmol/L during the third month (Fig 2B).

### Settlement succession of calcifying organisms

**Fig 3:**
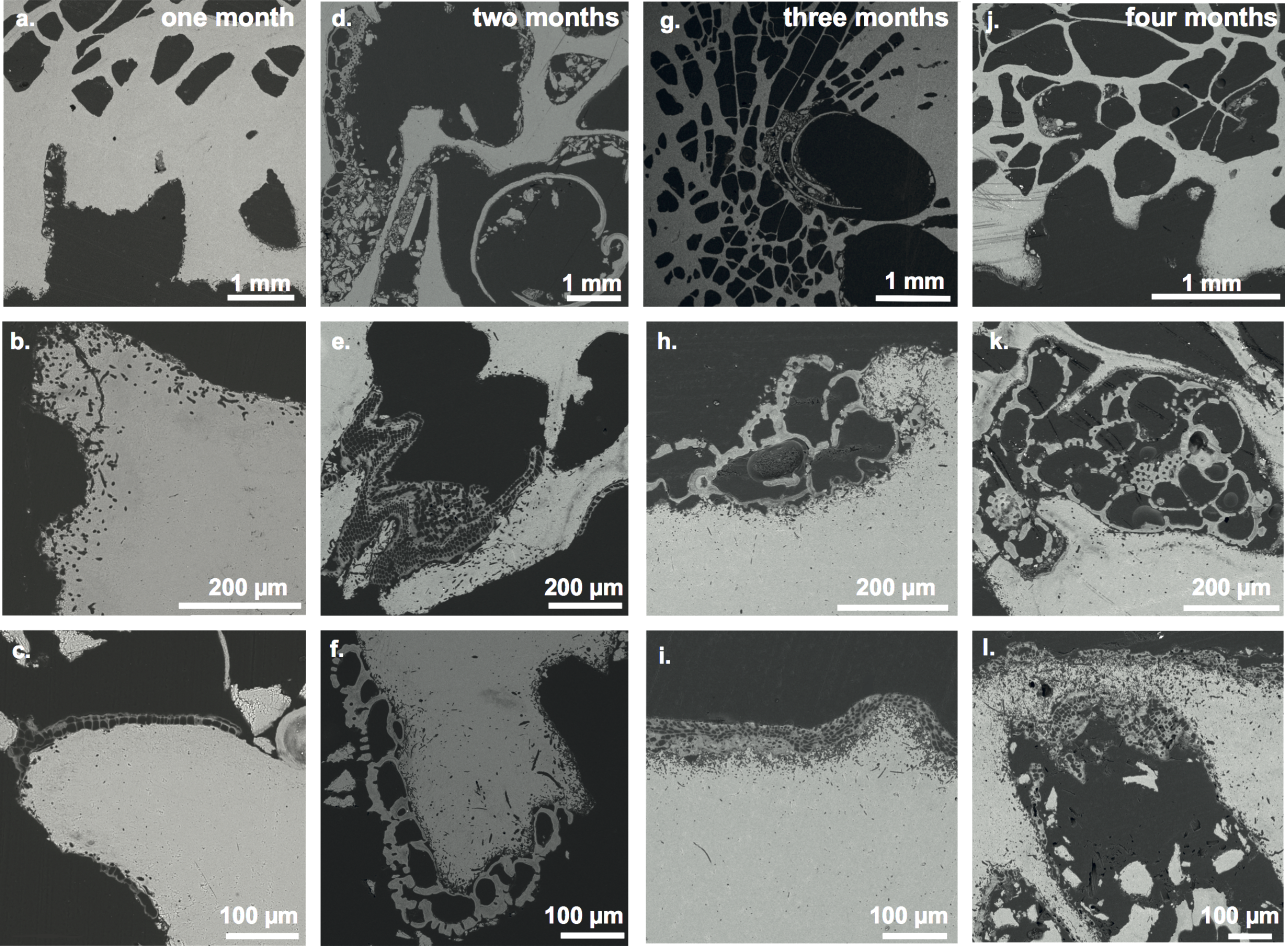
Time-series BSE images of thin-sections from coral substrates throughout the experiment. Shown are representative areas of thin-sections of coral substrates after a-c) one month, d-f) two months, g-i) three months, and j-l) four months of exposure. Encrusting species shown are c) crustose coralline red alga (CCA), d) lithophagine bivalve (genus *Lithophaga*/*Leiosolenus*), encrusting benthic foraminifer (*Homotrema rubrum*), e) encrusting bryozoan f) encrusting benthic foraminifer, g) lithophagine bivalve, h) encrusting benthic foraminifer, i) CCA, J) CCA (lower left) k) encrusting benthic foraminifer, and l) CCA. Note in k) darker thin bands indicate CaCO_3_ mineralogy change of the original coral skeleton (i.e. aragonite to calcite) due to microbioerosion. Also note the change in surface morphology and the increase in microbioerosion through time.

**Fig 4:**
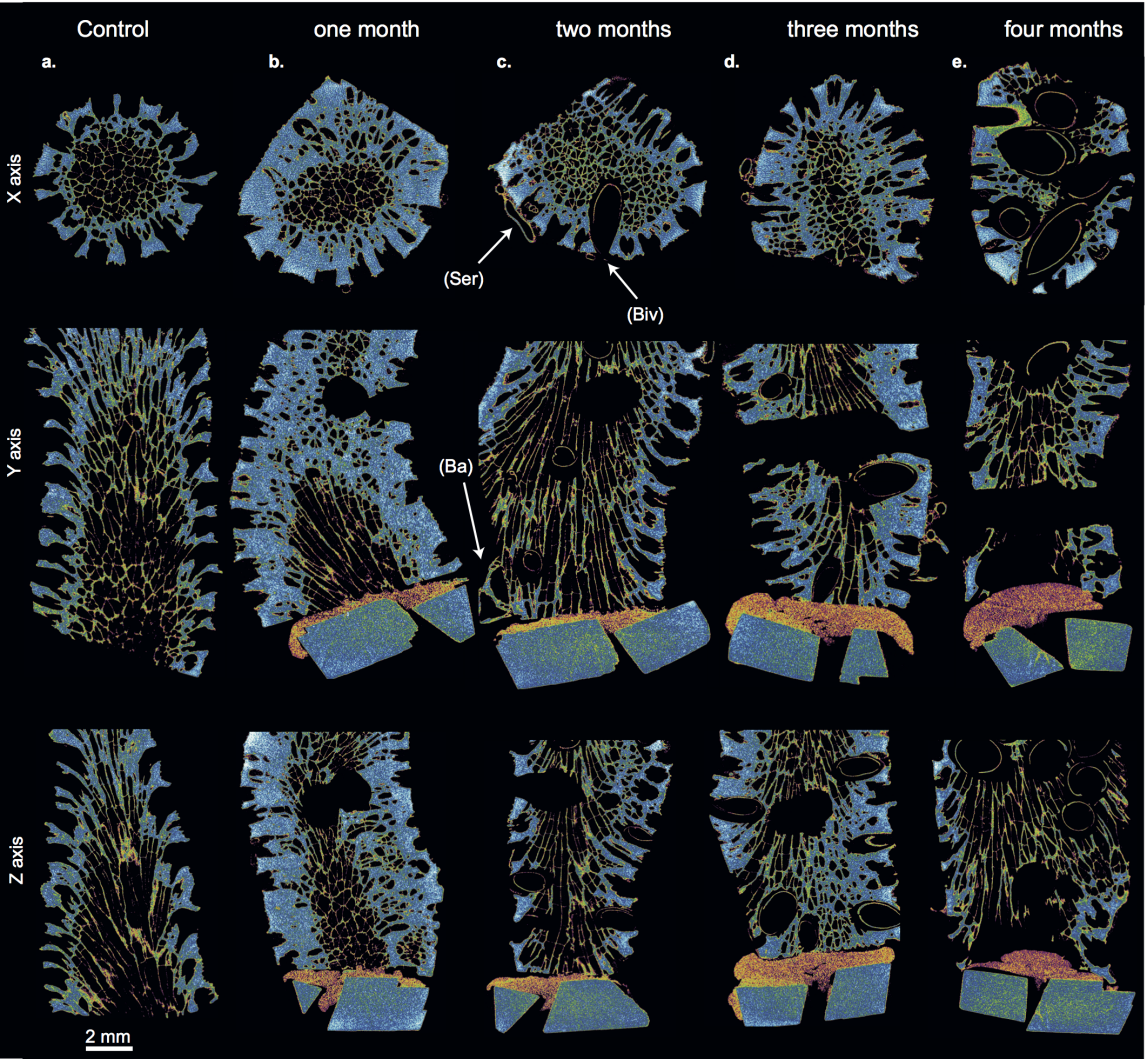
Cross sections from modeled μCT scans of substrates per exposure period, which indicate the settlement succession of the bioeroder community and the internal change in morphology. Shown are cross sections through the X-, Y- and Z-axis of coral substrates of a) control, and after b) one month, c) two months, d) three months, and e) four months of exposure. The hole in the middle part was pre-experimentally drilled to fix the substrates in the reef (cf. S1 Fig). Genera depicted in the μCT scan cross-sections are in c-e) serpulids (Ser), lithophagine bivalves (Biv), and balanids (Ba). Note the increase in abundance and size of lithophagine bivalves through time.

**Fig 5:**
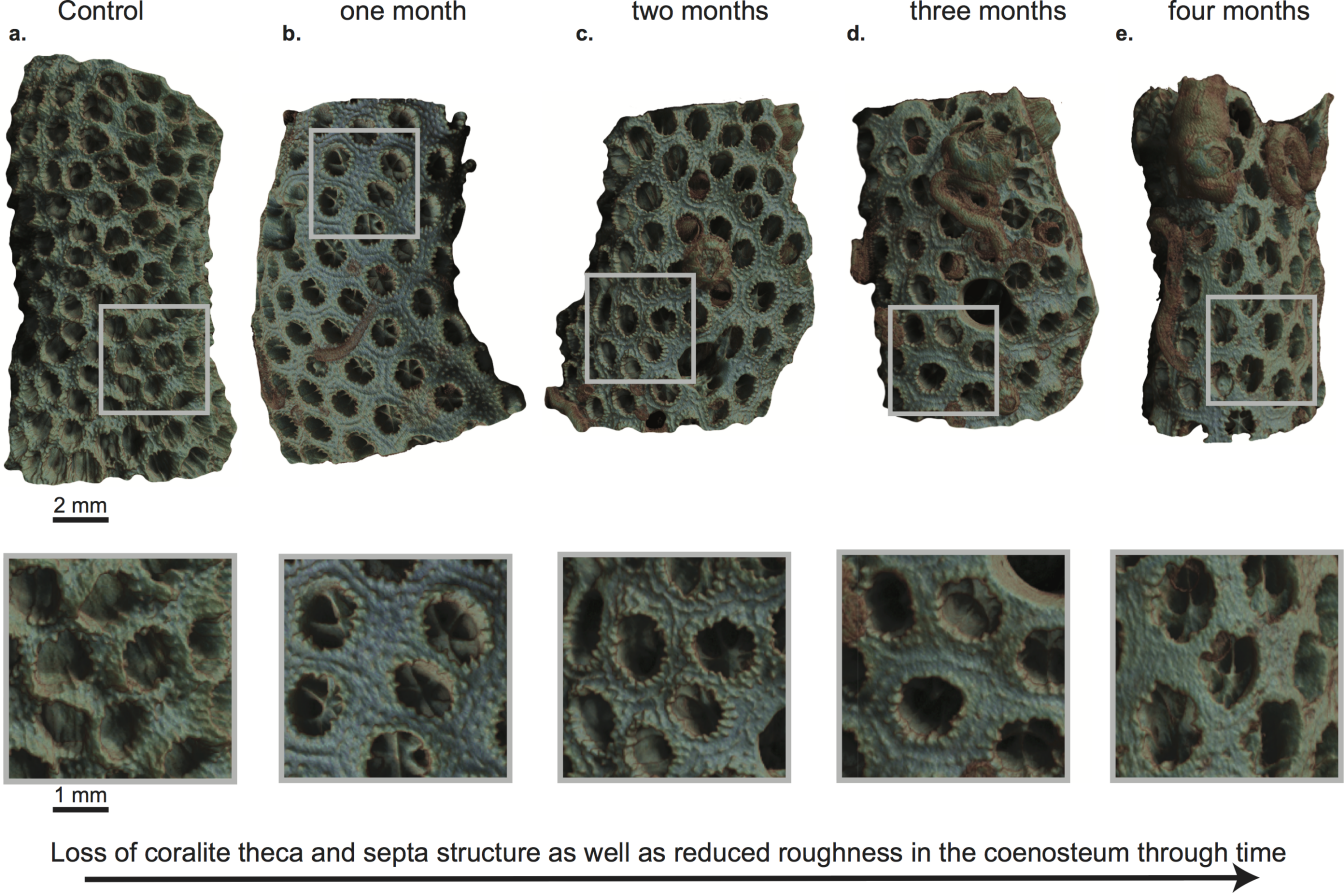
Modeled μCT scans showing the surface morphological change and the settlement succession of bioeroders on the coral substrates. Smaller quadrates at the bottom indicate the alteration of surface roughness per substrate and month. Shown are coral substrates of a) control, and after b) one month, c) two months, d) three months, and e) four months of exposure. Settled genera depicted are in b) serpulids and small CCA (lower left side), c) balanids and serpulids, d) balanids, serpulids and CCA (encrusting on right side, brownish color), and e) balanids and serpulids. Also see supplementary video files in S4–S8 Figs.

The calcifying community that developed inside and on the coral substrates consisted of phototrophic and organotrophic organisms. From μCT scans, photographs, and thin-sections the following calcifying genera were identified (Figs 3–5; S5–S9 Figs): crustose coralline red algae (CCA), biomineralizing polychaetes (serpulid worms), encrusting bryozoans, encrusting benthic foraminifers (*Homotrema rubrum*), lithophagine bivalves (S11 Fig, *Lithophaga* (*Leiosolenus)* cf. *aristata* (Dillwyn, 1817); [42], Leon Hoffmann, pers. comm.), and balanids (acorn barnacles). The settlement of the calcifiers followed a temporal trend. Crustose coralline red algae (CCA) and serpulid worms were primary settlers (present after one month; Figs 3C and 5B). Bryozoans and balanids were observed after two months, increasing in abundance with time of exposure (Figs 3C, 3D, 3E, 5B, 5C and 5D). Likewise, lithophagine bivalves were first observed after two months (Figs 3D and 4B). The number and size of the bivalves increased rapidly after three and four months of exposure (Fig 4D and 4E). However, reaching only 2 to 3 mm in size, the bivalves were still in a juvenile stage at the end of the experiment. The benthic foraminifer species *H*. *rubrum* was present from the second month onward (Fig 3D), encrusting the surface of the coral substrate between corallites (i.e. coenosteum) (Fig 3B, 3F, 3H and 3K).

### Macrobioerosion

The main macrobioeroder observed was the lithophagine bivalve, genus *Lithophaga*/*Leiosolenus* (S11 Fig). After two months of exposure, shells of these bivalves were identified in μCT scans inside the coral substrates (Figs 4 and 5; S6–S8 Figs). With increasing size and numbers of individuals through time, a substantial part of the internal CaCO_3_ coral substrate was bioeroded after the exposure period (Table 2; Figs 4 and 5; S8 Fig).

**Table 2:** Coral substrates deployed on December 3^rd^ 2013 at Matapalo Reef with date of collection, pre- and post-experimental weight, CaCO_3_ loss and indication, which individual substrates per exposure time are presented in Figures.

ID / Exposure	Collection date (d/m/y)	Pre-weight (mg)	Post-weight (mg)	CaCO_3_ loss (mg)	CaCO_3_ loss (%)	CaCO_3_ loss after months (mean %)	μCT and thin-section
37 / one month	06/01/2014	1474.5	1457.5	-17.0	1.15	0.87	
38 / one month	06/01/2014	2461.5	2445.5	-16.0	0.65		X
39 / one month	06/01/2014	1273.5	1266.3	-7.2	0.57		
40 / one month	06/01/2014	1875.6	1854.9	-20.7	1.10		
41 / two months	10.02.2014	2130.3	2116.2	-14.1	0.66	6.90	
42 / two months	10/02/2014	2257.9	1898.2	-359.7	15.93		
43 / two months	not recovered*	1491.7	-	-			
44 / two months	10/02/2014	1652.4	1584.5	-67.9	4.11		X
45 / three months	10/03/2014	1666.8	1593.2	-73.6	4.41	8.40	
46 / three months	10/03/2014	1510.0	1319.9	-190.1	12.58		
47 / three months	not recovered*	1520.2	-	-			
48 / three months	10/03/2014	2699.5	2478.1	-221.4	8.20		X
77 / four months	07/04/2014	1531.2	1048.9	-482.3	31.50	32.65	X
78 / four months	07/04/2014	1931.0	1278.5	-652.5	33.79		
79 / four months	not recovered*	1288.4	-	-			
80 / four months	not recovered*	1257.9	-	-			

*eroded by bioerosion, lost to the substratum

### Microbioerosion

**Fig 6:**
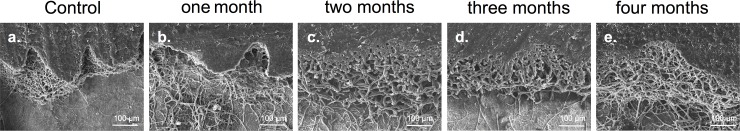
SEM images of cast-embedded and partially etched cross sections of coral substrates with positive infills of microbioerosion traces on the skeletal surface. Shown are coral substrates of a) control, and after b) one month, c) two months, d) three months, and e) four months of exposure. Most of the observed bioerosion traces were produced by euendolithic cyanobacteria complemented by some traces formed by chlorophyte algae and marine fungi. Note the increase in boring density over time and the increase in the depth of penetration into the skeletal structure.

By investigating microbioerosion traces in the epoxy resin casts of the control and exposed substrates, an increase in the diversity of microbioerosion became evident. SEM images of the surface of the control substrate show a comparatively intact original substrate structure (i.e. fine detail of coral fibers are visible; S3 Fig). Nevertheless, some degree of syn-vivo microbioerosion, mainly by the ubiquitous symbiotic chlorophyte algae *Ostreobium quekettii*, was present before the deployment of the substrates (Fig 6A). Traces of microbioeroders in the control substrate were predominantly located at the surface of the coenosteum, where polyp tissue cover is generally thinner in living specimens. Throughout the experiment the coral substrates became progressively altered by microbioeroders with an overall increase in average penetration depth (Fig 6, S3 Fig). Deep skeletal microbioerosion is typically enhanced when live polyp tissue is damaged or removed and active re-calcification of the coral ceases. The observed microbioerosion traces identify endolithic cyanobacteria as the main agents of microbioerosion during the experiment (complemented by some chlorophyte algae and marine fungi), while they were absent in the pre-experiment control sample (Fig 6). Since cyanobacteria and chlorophytes are phototrophs, the density of their bioerosion traces in the experimental substrates was governed by the orientation of the substrates, and hence light exposure, resulting in a heterogeneous distribution evident around the circumference of substrate cross sections. Traces of microborers reach the inner parts of the coral skeleton only in substrates retrieved after three and four months (Fig 6D and 6E).

### Abiotic CaCO_3_ cementation and mineralogy

BSE analyses of thin-sections from coral substrates did not show signs of early internal cementation of the skeletal structure (e.g. crystals of aragonite needles) after the four months exposure period (Fig 3A and 3J). No gross diagenetic alteration of the original aragonite coral skeleton was observed (i.e. coral fibers of the substrate preserved). BSE images show uniform mineralogy of the original coral skeleton (gray-scale value). However some local mineral recrystallization of the CaCO_3_ from aragonite to calcite adjacent to bioerosion traces was observed (cf. Fig 3K; areas with darker grey level within the coral skeleton), indicating micritization of the original coral skeleton.

### Changes in net bioerosion CaCO_3_ substrate budget

The time-series analysis of the net bioerosion CaCO_3_ substrate budget (accretion minus bioerosion) shows an overall negative trend with a mean loss of 0.5 ± 0.2 mg CaCO_3_ d^-1^ over the four months period of the experiment, which over the exposure period equates to a mean ~9% CaCO_3_ substrate loss per month (Fig 2C; Table 2). The one-way ANOVA results and post-hoc Tukey HSD indicate a highly significant loss of CaCO_3_ during the final month of exposure, after the onset of upwelling (*p* <0.01; Table 3, S1 Table). However, the statistical tests are based on very low replication and therefore demand cautious interpretation. The net CaCO_3_ loss per day increased from a rate of <0.5 mg d^-1^, for substrates exposed from one to three months, to a rate of >1 mg d^-1^, after the upwelling pulse. The mean net CaCO_3_ loss rate of the substrates that were sampled after the fourth month of exposure was ~1.5 mg CaCO_3_ d^-1^, which equates to a ~36% total CaCO_3_ loss of these substrates (Table 2). The substrate´s CaCO_3_ budget change (i.e. the strong increase in CaCO_3_ loss for substrates of four months of exposure) also correlates with a shift in settlement community. The community shift is represented by a change from phototrophic (e.g. CCA) to larger organotrophic calcifying genera (bivalves and barnacles) that settled especially during the last two months. Primarily, bioerosion from bivalves (genus *Lithophaga*/*Leiosolenus*) and microbioerosion caused net CaCO_3_ loss of original coral substrate (Figs 2C, 3L, 4D and 4E). However concerning the net bioerosion CaCO_3_ budget of the substrates this has to be viewed in the context that the calcifying organisms of the settlement community produce CaCO_3_ shells (i.e. may be reworked to consolidated carbonate sediment after death and thereby contribute to accretion of the reef platform). Thus, these calcifying settlers biased the total CaCO_3_ loss of the coral substrates, which in this study was not investigated separately.

**Table 3:** Analysis of variance from the exponential loss rate of CaCO_3_ mg d^-1^. Post-hoc Tukey HSD identified a significantly different rate of CaCO_3_ loss only in the final month of exposure, after the onset of upwelling (S1 Table). Note that statistical results base on low replication.

Source of Variance	DF	SS	Mean Square	F Ratio	Prob > F
Month	3	2.355	0.785	13.341	0.002
Error	8	0.471	0.059		
Total	11	2.826			

## Discussion

### Seawater characteristics at Matapalo Reef

Low seawater temperature, low dissolved oxygen concentration and enhanced nutrient concentrations provide evidence that several cold water intrusions (i.e. upwelling event) influenced the study site at Matapalo Reef during the period from December 2013 to April 2014 [23,24]. Data from Marina Papagayo (a field site within ~200 m distance to a coral reef) showed that increased intrusions of cold and nutrient-enriched subsurface water rised seawater *p*CO_2_, lowered pH and decreased Ω_arag_ [37,39]. As indicated by these data, pH and Ω_arag_ generally decreased concordantly with lower seawater temperatures, reflecting a strong influence of the corrosive subsurface (i.e. upwelled) waters on the seawater carbon chemistry at Marina Papagayo (S12A and S12B Fig). Compared to these trends the mean pH_VINDTA_ and Ω_arag_ derived from the A_T_ and DIC measurements at Matapalo Reef are enhanced during upwelling. This means that at the measured seawater temperatures one would expect a much lower pH and Ω_arag_, given the fact that A_T_/DIC ratio controls pH and Ω_arag_ and an increasing A_T_/DIC ratio raises both the pH and Ω_arag_ (S12C Fig). Photosynthesis production of organic matter, and the dissolution of CaCO_3_ are two processes increasing the A_T_/ DIC ratio. The elevated pH and Ω_arag_ at Matapalo Reef (i.e. when compared to the seawater temperature, and to measurements at Marina Papagayo) could accordingly be explained by a stronger response of photoautotrophic organisms and bioeroders to the intrusion of corrosive and nutrient-enriched seawater. Such an amplified response to the intrusion of cold subsurface water could also explain why the pH did not drop during the main upwelling event in February 2014. The reason why CaCO_3_ dissolution can occur despite CaCO_3_ over-saturation is that the conditions measured in the water column likely differ from conditions at the substrate-seawater interface (i.e. diffusive boundary layer effect; e.g. [43]). Seawater within the boundary layer of the substrate-seawater interface may well be CaCO_3_ under-saturated due to activity of the settlement community creating erosive conditions. By dissolution of CaCO_3_ substrate, bioerosive activity may have caused a carbonate buffer effect of the surrounding seawater covering the reef benthos (i.e. assumingly a phenomenon ranging few meters in the water column, depending on currents), which is reflected in the measured seawater parameters (i.e. elevated seawater pH and Ω_arag_).

### Successive calcareous macrobioeroder community settlement

Due to the specific environmental conditions in the ETP reefs, it is known that the temporal succession of macroborer communities differs from trends observed in reefs less influenced by upwelling [44]. The rapid development of the settlement community indicates high larvae abundance in the reef during the upwelling period, with environmental conditions beneficial for macrobioeroders. Serpulids and bryozoans are considered to be opportunistic colonizers in the initial stage of substrate infestation [45,46], whereas for lithophagine bivalves such an early succession is unusual [42]. In typical tropical reef settings, lithophagine bivalves are first observed after one year or even longer time periods, thus in a much later successional stage [47–50]. In our experiment, the bivalves represent the most prominent group of macrobioeroders. The use of natural coral substrate likely benefited the rapid settlement observed, compared to the use of CaCO_3_ blocks, and thus may represent a realistic scenario of sedimentary infestation. The skeletal morphology of the coral substrate used is comparable with the *Pocillopora* coral rubble at the study site (e.g. corallite size; [34]). Bivalve veliger larvae likely entered the coral substrate through calices and between septae, as other lithophagines do also in live corals [51]. No boreholes from bivalves were found at the coenosteum. However, lithophagine bivalves boring into live coral tissue may not be this rapid when polyps are present (i.e. defense mechanisms of the coral; [52]). Infestation and fragmentation of living coral branches by lithophagine bivalves can support coral dispersal [6,53]. In reefs off Panama, intense settlement of lithophagine bivalves was observed during upwelling conditions. During the non-upwelling season almost no recruitment of bivalve larvae was observed [42].

### CaCO_3_ cementation

Abiotic precipitation of secondary CaCO_3_ cements was not observed during the four months exposure period. Although Matapalo Reef is a relatively sheltered near-shore environment, it does experience a relatively low seawater CaCO_3_ saturation state (Ω_arag_ <3; [22,39,54]. This may suggest that the low Ω_arag_ is a cause for the lack of secondary CaCO_3_ cements [27]. Moreover, the settlement community likely lowers Ω_arag_ further at the substrate-seawater interface. In marginal reef environments with comparatively poorly developed reef framework, similar to the present study site, an envelope of encrusting calcifiers (e.g. CCA, encrusting benthic foraminifers, serpulids, and barnacles) fills the role of stabilizing the reef framework [7,15]. Despite bio-corrosive alteration of the skeletal substrate structure, a gross change in mineralogy (e.g. aragonite to calcite, or crystal structure alteration) was not observed. However, minor CaCO_3_ recrystallization (from aragonite to calcite) and micritization of the original coral skeleton was present in close vicinity to microborings (cf. Fig 3K). This was likely caused by the metabolism, exudates and acidic substances of the (micro) bioeroder community.

### CaCO_3_ erosion and dissolution

When considering the whole exposure period, bivalves of the genus *Lithophaga*/*Leiosolenus* are the main macrobioeroders of CaCO_3_ coral substrate. Bivalve boreholes increased in size and abundance with increased exposure time, which resulted in a marked increase in CaCO_3_ substrate loss especially during the upwelling months (Feb/Mar). Another important cause for the rapid CaCO_3_ substrate loss through time is endolithic microbioerosion. Number and penetration depth of microbioerosion traces also increased considerably with exposure time (cf. Figs 3 and 6; S3 Fig). Substrates of the last two months (Feb/Mar) show a gradual morphological degradation of the corallite microstructure and the coenosteum (including the papillae), which consequently may be a further result of the progressive increase of microbioerosion on the exposed surface (Figs 5 and 6; S3 Fig).

The observed CaCO_3_ recrystallization associated with the bioeroder community indicates that CaCO_3_ dissolution likely is biologically mediated. Besides chemically-based CaCO_3_ bioerosion by some species of bioeroders, the dissolution of coral substrate skeleton may also originate from physiologically mediated alteration of the diffusive boundary layer conditions through the settling organisms, which may have created seawater CaCO_3_ under-saturation (Ω_arag_ <1) at the substrate-seawater interface. This assumption is supported by the fact that the onset of intense CaCO_3_ substrate loss correlates with enhanced settlement of organotrophic species such as serpulids, bryozoans, barnacles, lithophagine bivalves (i.e. metabolic respiration) from the second month onward, favored by elevated nutrient conditions with the onset of upwelling. To a minor part bioerosive grazing and predation (e.g. of mollusks, crustaceans, echinoderms, reef fish) may have contributed to the observed erosion pattern. The complete loss of some substrates, especially for the substrates exposed for four months, may well be complete crumbling due to external and internal bioerosion, the lack of intragranular cementation and sufficient external encrustation.

### Net bioerosion CaCO_3_ budget change

The coral substrates underwent a significant CaCO_3_ loss of ~36% total dry weight after four months of exposure. This resembles a mean loss of >1 mg CaCO_3_ d^-1^ in coral substrates that were exposed in the reef for the whole experimental period (Fig 2C, Table 2). However, the mean CaCO_3_ loss per day was significantly higher in the substrates exposed for four months compared to the substrates exposed only up to three months (<0.5 mg CaCO_3_ d^-1^), which indicates an enhancement of the bioerosive activity during the fourth month and after the onset of upwelling (p <0.01; Fig 2, Table 2; S1 Table). When additionally considering the possibility of crumbling of the lost substrates exposed for four months, total CaCO_3_ loss even exceeded 50%. It has to be noted that these time-series results on the bioerosion of the substrate CaCO_3_ budget are based on low replication, i.e. local representatives in a patchy reef environment. Spatially larger-scaled studies are needed to validate the observed trend for the influence of bioerosion on the CaCO_3_ budget in ETP reefs.

**Fig 7:**
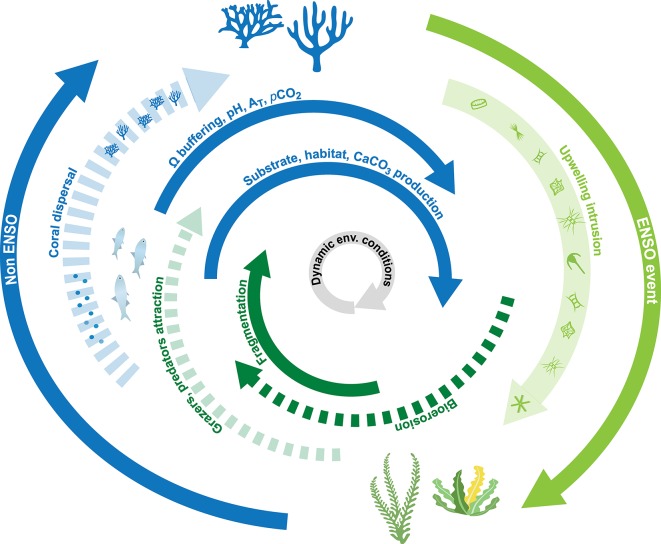
Graphical concept of the role of bioerosion in ETP coral reef community transitions. Short-term transitions between coral and algal dominance can occur due to changes in environmental boundary conditions. Coral growth may cease during ENSO events or during periods of intensive upwelling. Eutrophic conditions in the reef favor organotrophic settlers, in particular detritus and filter feeders, including many calcifying encrusting and bioeroding species. Enhanced activity of bioeroders, as an ecological response, supports the re-transition of the reef into an oligotrophic condition by the uptake of nutrients and buffering seawater carbon chemistry (carbonate sediment production and dissolution; influence seawater Ω, pH, A_T_, *p*CO_2_). Additionally, grazers and predators are attracted due to the increase in food or prey abundance. New substrate is formed by predation (reef fish, echinoderms, mollusks) and macrobioerosion, which allows coral dispersal (fragmentation) and formation of rhodolithic substrate serving as larvae settling grounds. The ecological effects benefit the growth of phototrophic calcifiers (i.e. corals, crustose coralline algae). Dashed lines indicate ecological responses to environmental processes (solid lines). Green lines indicate an effect on the reef community towards algal growth and blue lines indicate effects towards coral growth.

The organisms of the encruster and macrobioeroder community build CaCO_3_ skeletons and shells that contribute to the carbonate sediments. In addition to corals, these organisms are also an important source of CaCO_3_ for the reef ecosystem and thus both negatively and positively contribute to the CaCO_3_ budget of the reef. The production of CaCO_3_ by these organisms may be especially important for the reef´s CaCO_3_ budget during periods with disruptive environmental events, when coral growth may cease (cf. Fig 7, eutrophic condition; [24]). Interestingly, the observed loss in CaCO_3_ substrate may explain the elevated pH and Ω_arag_ at Matapalo Reef (i.e. when correlated to the seawater temperature). This indicates an effect of bioerosion on the carbonate buffer capacity of the seawater (Table 1, Fig 2A, S12 Fig). If so, bioerosion causing CaCO_3_ dissolution (e.g. of coral rubble substrate; cf. S2 Fig) may on the one hand thrive under the high *p*CO_2_ conditions associated with upwelling. On the other hand however, bioerosion-driven CaCO_3_ dissolution may aid to mitigate effects of the upwelled corrosive seawater on reef health (i.e. local carbonate buffer against abiotic dissolution of the living reef framework).

### Bioerosion and encrustation under dynamic environmental conditions and their role for ecosystem functioning in ETP coral reefs

Bioerosion rates in ETP reefs are among the highest recorded in the world [31,55,56]. The rapid macro- and microbioerosion observed at Matapalo Reef confirms previous investigations. Variable boundary conditions and ENSO events can cause ETP reefs to experience environmental transitions with temporary die-off and re-growth of corals (Fig 7) [57]. During periods of intense upwelling with high nutrient concentrations, reef ecosystems may become algal dominated (which at Matapalo Reef is the fleshy green algae of the genus *Caulerpa;* S2 Fig) that negatively affects coral growth [23,24,58]. Additionally, with the onset of upwelling, bioerosion on corals increases. This facilitates the creation of unique habitats besides the coral reef community, like the cryptic coral rubble habitat [59]. The species that live within this habitat originate from different environmental and oceanographic regimes and form “historically-developed” communities in ETP reefs [60]. These communities that consist primarily of eroders and encrusters influence the reef´s resilience by triggering various environmental responses, e.g. the attraction of predators and grazers, coral dispersal and the formation of new substrate. Consequently, the evoked effects may allow the reef ecosystem to regain oligotrophic conditions that benefit coral growth (Fig 7) [61]. It is a well-known ecological principle that in ecosystems under (temporal) environmental stress, biological processes promoting regeneration capacity gain momentum [62–64].

Especially in marginal reefs of the ETP, such transitions may occur frequently due to disruptive environmental events, resulting in periods of stagnation and (re-)commencement of coral growth [23,24]. The main parameters known to steer the cyclicality of coral die-off events are varying oceanic boundary conditions (e.g. El Niño/La Niña events) [65,66]. Other possible synergistic causes include predator/prey relationships, grazer abundance or diseases, and climate change [23,24,58]. While influences from the land or human made pollution are not yet a major factor, they may become more prominent in the future [67]. The question is, how resilient these reefs will be under future climate change scenarios? However, “historically-developed” and interconnected community structures in ETP reefs may still enable them to recover after temporal environmental stress.

## Conclusion

In this study, we present the rapid development and alternate succession of an encruster and bioeroder community on coral substrate in an ETP coral reef. Foremost the rapid settlement of lithophagine bivalves as the main macrobioeroders of the substrates is particular to coral reefs in the ETP. CaCO_3_ erosion of the substrate by bioeroders increased markedly with the onset of upwelling. Derived from our time-series experiment, bioerosion caused a negative CaCO_3_ substrate budget. Dissolution of CaCO_3_ agrees with the elevated Ω_arag_ and pH observed at Matapalo Reef, when compared to the site at Marina Papagayo, which is located in ~200 m distance to a reef. The resulting local carbonate buffer effect favored an influx of atmospheric CO_2_ into reef waters. This may suggest that even in upwelling influenced reef zones, ocean waters are still capable to take up atmospheric CO_2_, and presently mitigate and conceal the global concentration rise caused by anthropogenic sources.

For the ecosystem scope, the settlement community provides important functions, such as habitat formation, and substrate consolidation. The community may even have an effect on the reef´s seawater carbon chemistry, enhancing the carbonate buffer capacity. With these functions settlement communities give plasticity to marginal coral reefs where dynamic environmental conditions, such as upwelling, can temporarily impair coral growth. The rapid bioerosion observed in ETP reefs thus provides a possible future scenario for tropical coral reefs affected by ocean acidification and eutrophication. Up to now, encrusting and bioeroding organisms complement the resilience potential of marginal reefs as an important part of their “historically-developed” community structures. However, these communities will likely become altered due to climate change, and marginal reef ecosystems may become locked in eutrophic, bioerosive conditions. The resulting negative CaCO_3_ substrate budget due to enhanced bioerosion, paired with the absence of secondary cementation, may have negative consequences for net reef accretion. However, if marginal reef ecosystems are protected from further and upcoming anthropogenic impacts and are granted sufficient time to recover, natural regeneration processes stimulated by settlement communities of encrusting and bioeroding organisms may still assist in the remediation of such temporarily stressed coral reefs.

## Supporting information

S1 Table Post-hoc Tukey HSD, Ordered differences report. (DOCX)

S2 Table Data comparison of measured and calculated seawater parameters from bottle samples (VINDTA) and Manta multiprobe. (DOCX)

S1 Fig Experimental setup deployed in the reef (schematic drawing and photograph). (JPG)

S2 Fig Photographs of typical benthic seafloor cover and sediment at the Matapalo Reef site. Crustose coralline red algae (CCA) encrusting the coral rubble substrate forming rhodoliths, and growth of the green macro-alga genus *Caulerpa*. Water depth ~5 m bsl.
(JPG)

S3 Fig SEM images from microbioerosion traces on the surface of the coral substrates. a) control, and after b) one month, c) two months, d) three months, and e) four month of exposure. Note the increase in borings and the loss of skeletal structure (e.g. coral fibers) over time. Scale bar 50 μm.
(TIFF)

S4 Fig μCT scan video of control coral substrate (pre-experiment). (MKV)

S5 Fig μCT scan video of coral substrate exposed for one month at Matapalo Reef. (MKV)

S6 Fig μCT scan video of coral substrate exposed for two months at Matapalo Reef. (MKV)

S7 Fig μCT scan video of coral substrate exposed for three months at Matapalo Reef. (MKV)

S8 Fig μCT scan video of coral substrate exposed for four months at Matapalo Reef. (MKV)

S9 Fig Photographs of retrieved coral substrates from the Matapalo Reef site, before bleaching (30% H2O2). After a, b) one month; c, d) two months; e, f) three months; g, h) four months of exposure.
(JPG)

S10 Fig Video showing the reconstructed μCT scan of one coral substrate (example) retrieved after two months of exposure from the field site Bahía Santa Elena. Note the enhanced settlement of balanids (acorn barnacles), i.e. competition for space, and the presence of lithophagine bivalves.
(MKV)

S11 Fig SEM images of the internal and external valve from one lithophagine bivalve, juvenile stage, identified as *Lithophaga* (*Leiosolenus*) cf. *aristata* (Dillwyn, 1817). Scale bar 500 μm.
(TIFF)

S12 Fig Graphs showing the correlation between measurement period means of a) pH and temperature, b) Ω_arag_ and temperature, and c) Ω_arag_ and A_T_/DIC at Marina Papagayo (black dots; 2009, 2012, and 2013; data from [37,39]) compared to the study site at Matapalo Reef (black squares; 2013/2014; data from [23], this study). Regression lines exclude data from Matapalo Reef. At Matapalo Reef, seawater pH and Ω_arag_ are elevated.
(EPS)
