# Supplementary material for: Rapid bioerosion in a tropical upwelling coral reef
Source: PLoS One. 2018 Sep 12;13(9):e0202887. doi: 10.1371/journal.pone.0202887 (PMC6135564; doi:10.1371/journal.pone.0202887)
Supplement: S1 Table — (DOCX) [file pone.0202887.s001.docx]

**S1 Table** Ordered Differences Report, m=months of exposure

| **Level** | **- Level** | **Difference** | **Std Err Dif** | **Lower CL** | **Upper CL** | **p-Value** |
| --- | --- | --- | --- | --- | --- | --- |
| 1m | 4m | 1,307603 | 0,2100823 | 0,634847 | 1,980359 | 0,0011* |
| 2m | 4m | 1,038740 | 0,2214462 | 0,329593 | 1,747887 | 0,0068* |
| 3m | 4m | 1,020994 | 0,2214462 | 0,311848 | 1,730141 | 0,0075* |
| 1m | 3m | 0,286608 | 0,1852752 | -0,306707 | 0,879923 | 0,4562 |
| 1m | 2m | 0,268863 | 0,1852752 | -0,324452 | 0,862178 | 0,5056 |
| 2m | 3m | 0,017745 | 0,1980675 | -0,616535 | 0,652025 | 0,9997 |
