# Supplementary material for: Rapid bioerosion in a tropical upwelling coral reef
Source: PLoS One. 2018 Sep 12;13(9):e0202887. doi: 10.1371/journal.pone.0202887 (PMC6135564; doi:10.1371/journal.pone.0202887)
Supplement: S2 Table — (DOCX) [file pone.0202887.s002.docx]

| **Date_VINDTA_** | **Time** | **Depth (m)** | **A_T_ (µmol/kg)** | **DIC (µmol/kg)** | **SST (°C)** | **SSS** | **pH-cal tot** | **fCO_2_-cal (µatm)** | **Omega_arag_-cal** | **Date_Manta_** | **start** | **end** | **Temp (°C)** | **pH (tot)** | **Salinity** |
| --- | --- | --- | --- | --- | --- | --- | --- | --- | --- | --- | --- | --- | --- | --- | --- |
| 02/12/2013 | 16:10 | 6,00 | 2211,18 | 1971,99 | 25,63 | 32,52 | 7,97 | 479,48 | 2,81 | 12/2/2013 | 09:15 | 15:30 | 25,40 | 8,07 | 32,55 |
| 09/12/2013 | 15:30 | 6,00 | 2106,38 | 1805,37 | 27,87 | 31,08 | 8,09 | 330,82 | 3,49 | 12/9/2013 | 09:15 | 15:00 | 27,97 | 8,09 | 31,03 |
| 16/12/2013 | 15:30 | 6,00 | 2093,72 | 1822,51 | 28,20 | 31,00 | 8,03 | 385,15 | 3,18 | 12/16/2013 | 15:45 | 17:30 | 28,40 | 8,06 | 30,86 |
| 23/12/2013 | 15:30 | 6,00 | 2072,18 | 1785,09 | 28,16 | 30,68 | 8,07 | 343,27 | 3,35 | 12/23/2013 | 09:30 | 15:00 | 28,45 | 8,09 | 30,60 |
| 30/12/2013 | 15:30 | 6,00 | 2078,75 | 1783,16 | 28,53 | 30,70 | 8,08 | 335,76 | 3,45 | 12/30/2013 | 15:30 | 18:00 | 28,63 | 8,11 | 30,69 |
| 06/01/2014 | 14:30 | 5,00 | 2086,62 | 1789,08 | 28,51 | 31,19 | 8,07 | 339,57 | 3,45 | 1/6/2014 | 09:00 | 15:00 | 28,85 | 8,12 | 31,07 |
|  |  |  |  |  |  |  |  |  |  | 1/14/2014 | 13:15 | 17:00 | 28,00 | 8,13 | 32,97 |
| 20/01/2014 | 13:13 | 3,25 | 2213,52 | 1890,27 | 26,41 | 31,92 | 8,12 | 318,66 | 3,73 | 1/20/2014 | 09:00 | 16:00 | 26,55 | 8,12 | 33,31 |
| 21/01/2014 | 08:30 | 3,25 | 2218,88 | 1918,48 | 26,41 | 30,54 | 8,10 | 345,66 | 3,55 |  |  |  |  |  |  |
| 23/01/2014 | 11:23 | 3,25 | 2209,45 | 1917,96 | 26,64 | 33,31 | 8,04 | 391,36 | 3,34 |  |  |  |  |  |  |
| 24/01/2014 | 13:00 | 3,25 | 2224,72 | 1938,93 | 25,98 | 32,02 | 8,06 | 381,87 | 3,33 |  |  |  |  |  |  |
| 25/01/2014 | 11:45 | 3,25 | 2207,52 | 1903,20 | 26,80 | 30,50 | 8,10 | 340,08 | 3,59 |  |  |  |  |  |  |
| 26/01/2014 | 12:15 | 3,25 | 2169,64 | 1864,63 | 27,46 | 32,98 | 8,06 | 360,27 | 3,49 |  |  |  |  |  |  |
| 27/01/2014 | 12:30 | 3,25 | 2185,60 | 1874,89 | 27,19 | 33,13 | 8,07 | 353,02 | 3,55 | 27/01/2014 | 09:00 | 14:15 | 27,17 | 8,10 | 33,20 |
| 28/01/2014 | 08:50 | 3,25 | 2198,65 | 1915,50 | 27,00 | 33,90 | 8,02 | 412,88 | 3,24 |  |  |  |  |  |  |
| 03/02/2014 | 12:30 | 5,50 | 2179,02 | 1889,40 | 27,00 | 33,65 | 8,03 | 390,48 | 3,30 | 2/3/2014 | 11:12 | 15:12 | 27,14 | 8,12 | 32,97 |
|  |  |  |  |  |  |  |  |  |  | 2/10/2014 | 14:00 | 17:00 | 23,64 | 8,30 | 33,83 |
|  |  |  |  |  |  |  |  |  |  | 2/17/2014 | 09:00 | 15:30 | 21,62 | 8,26 | 34,19 |
|  |  |  |  |  |  |  |  |  |  | 2/24/2014 | 15:30 | 17:00 | 25,03 | 8,29 | 33,58 |
|  |  |  |  |  |  |  |  |  |  | 3/3/2014 | 11:00 | 16:00 | 24,07 | 8,26 | 33,60 |
|  |  |  |  |  |  |  |  |  |  | 3/10/2014 | 09:00 | 15:00 | 28,17 | 8,16 | 32,87 |
|  |  |  |  |  |  |  |  |  |  | 3/17/2014 | 09:00 | 15:00 | 27,44 | 8,14 | 33,57 |
|  |  |  |  |  |  |  |  |  |  | 3/24/2014 | 09:00 | 14:30 | 26,62 | 8,05 | 33,52 |
| 31/03/2014 | 12:28 | 3,00 | 2256,14 | 1924,72 | 25,40 | 33,67 | 8,11 | 337,41 | 3,75 | 31/03/2014 | 11:00 | 15:45 | 25,67 | 8,13 | 33,65 |
|  |  |  |  |  |  |  |  |  |  | 4/7/2014 | 09:00 | 14:00 | 28,11 | 8,03 | 33,70 |
|  |  |  |  |  |  |  |  |  |  | 4/14/2014 | 13:30 | 17:00 | 28,23 | 8,07 | 33,59 |
| 17/04/2014 | 10:02 | 2,25 | 2263,01 | 1956,66 | 27,95 | 33,64 | 8,03 | 404,54 | 3,55 | 17/04/2014 | 08:00 | 10:30 | 28,23 | 8,07 | 33,62 |

Temp

pH_VINDTA_

pH_Manta_

Temperature

pH

Date
