# Supplementary figures and images for: Rapid bioerosion in a tropical upwelling coral reef
Source: PLoS One. 2018 Sep 12;13(9):e0202887. doi: 10.1371/journal.pone.0202887 (PMC6135564; doi:10.1371/journal.pone.0202887)

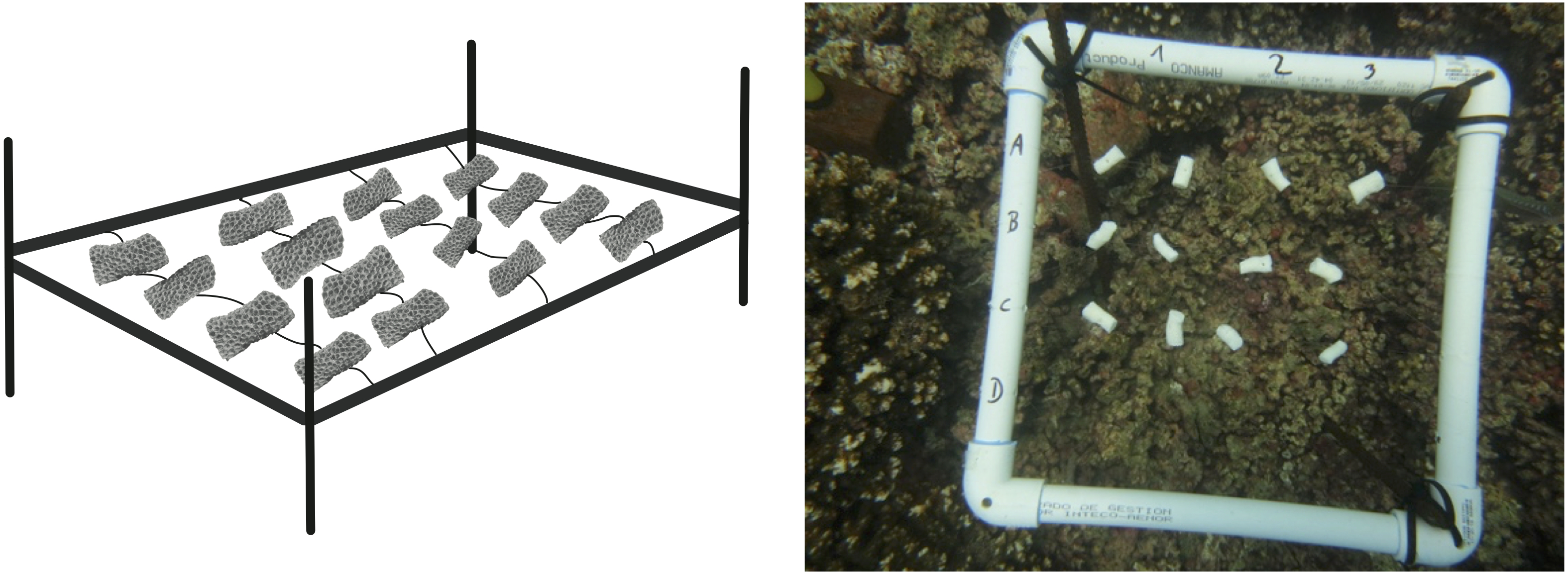

Supplement: S1 Fig — (JPG) [file pone.0202887.s003.jpg]

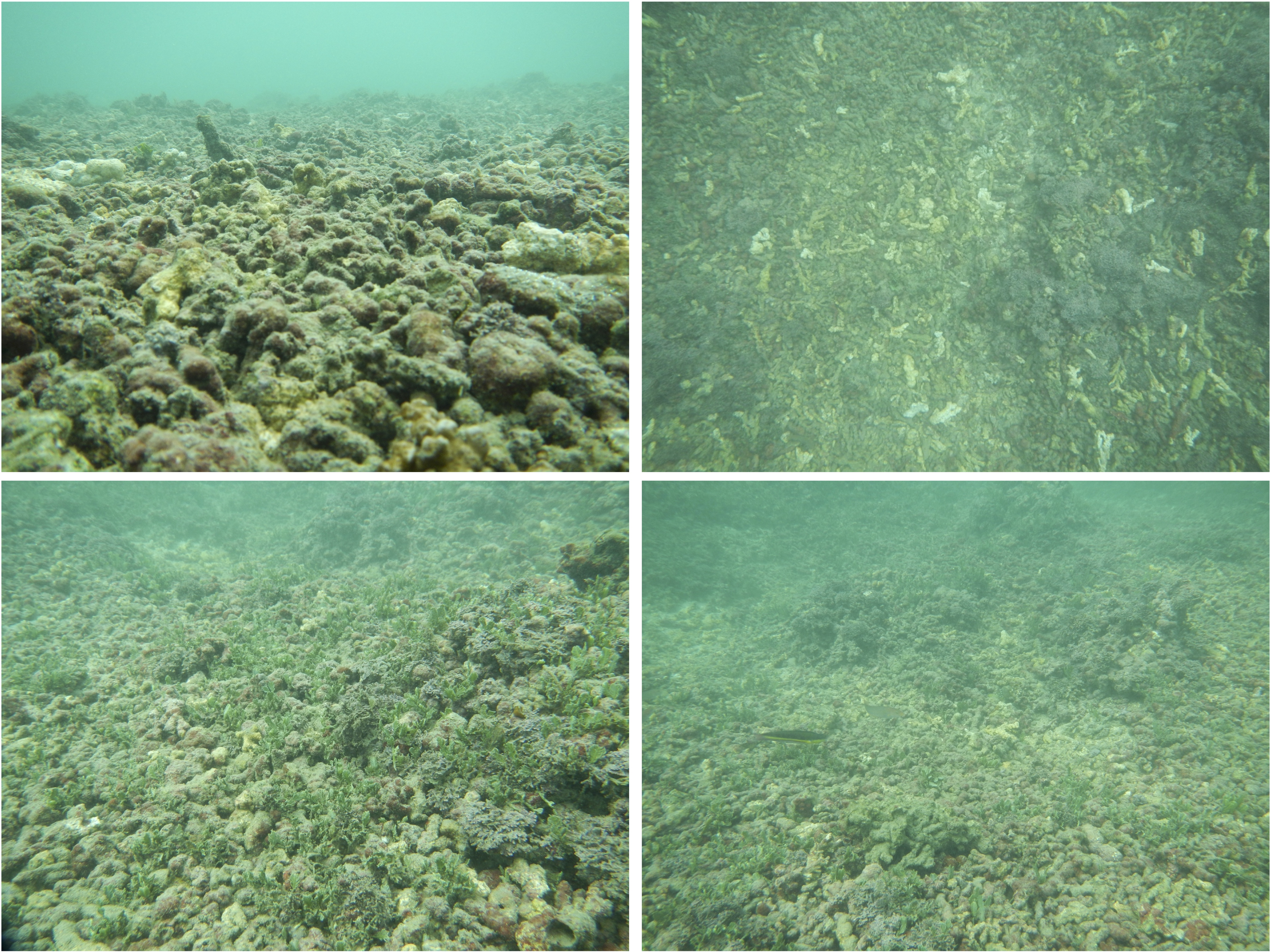

Supplement: S2 Fig — Crustose coralline red algae (CCA) encrusting the coral rubble substrate forming rhodoliths, and growth of the green macro-alga genus Caulerpa. Water depth ~5 m bsl. (JPG) [file pone.0202887.s004.jpg]

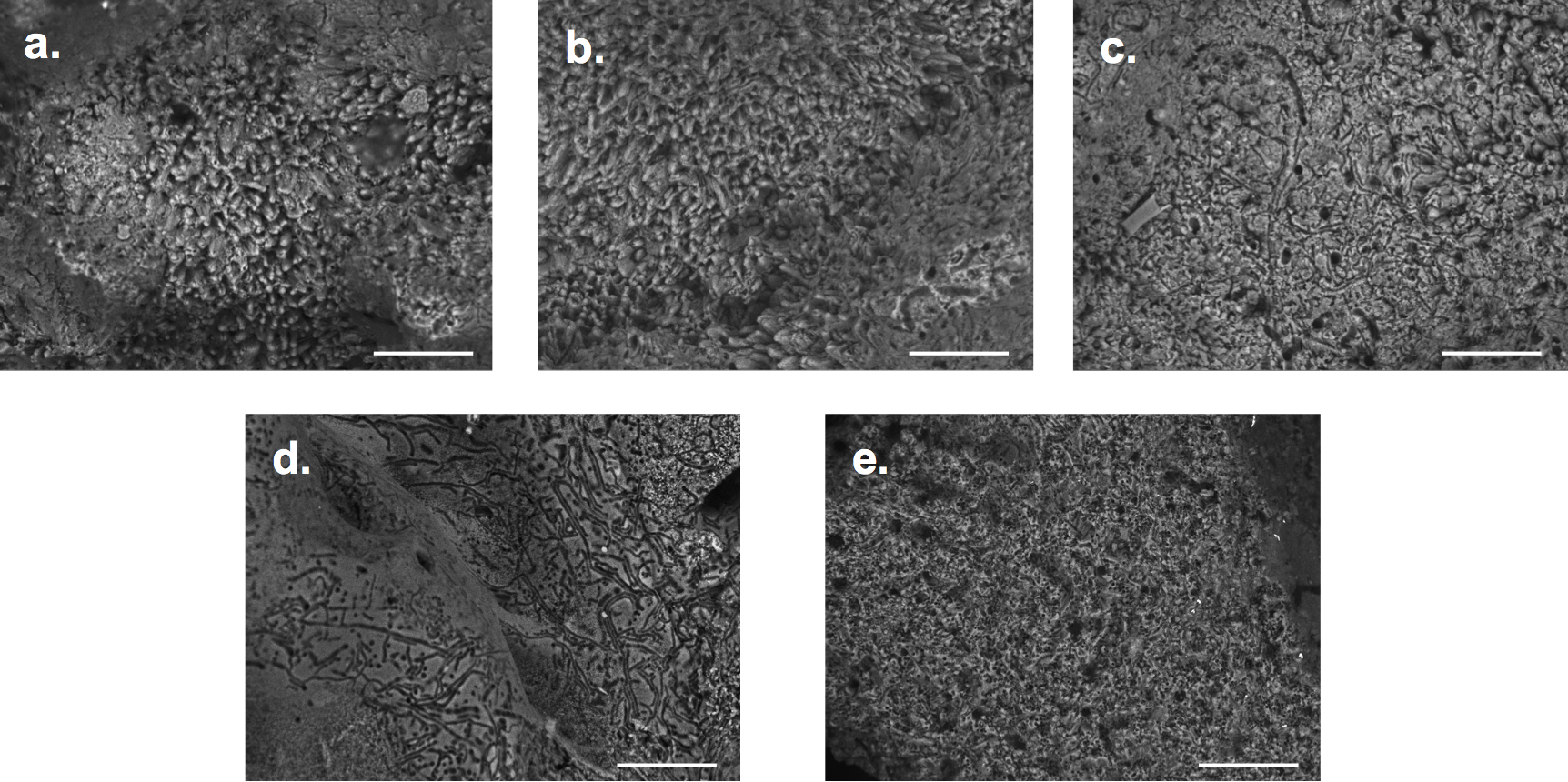

Supplement: S3 Fig — a) control, and after b) one month, c) two months, d) three months, and e) four month of exposure. Note the increase in borings and the loss of skeletal structure (e.g. coral fibers) over time. Scale bar 50 μm. (TIFF) [file pone.0202887.s005.tiff]

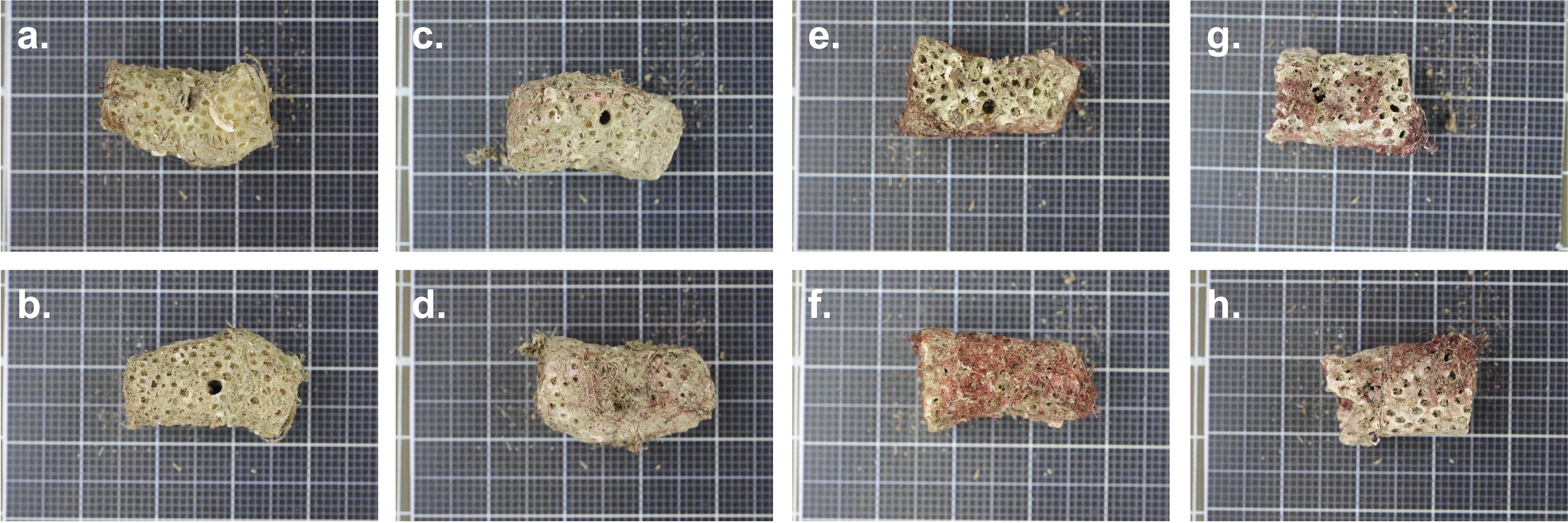

Supplement: S9 Fig — After a, b) one month; c, d) two months; e, f) three months; g, h) four months of exposure. (JPG) [file pone.0202887.s011.jpg]

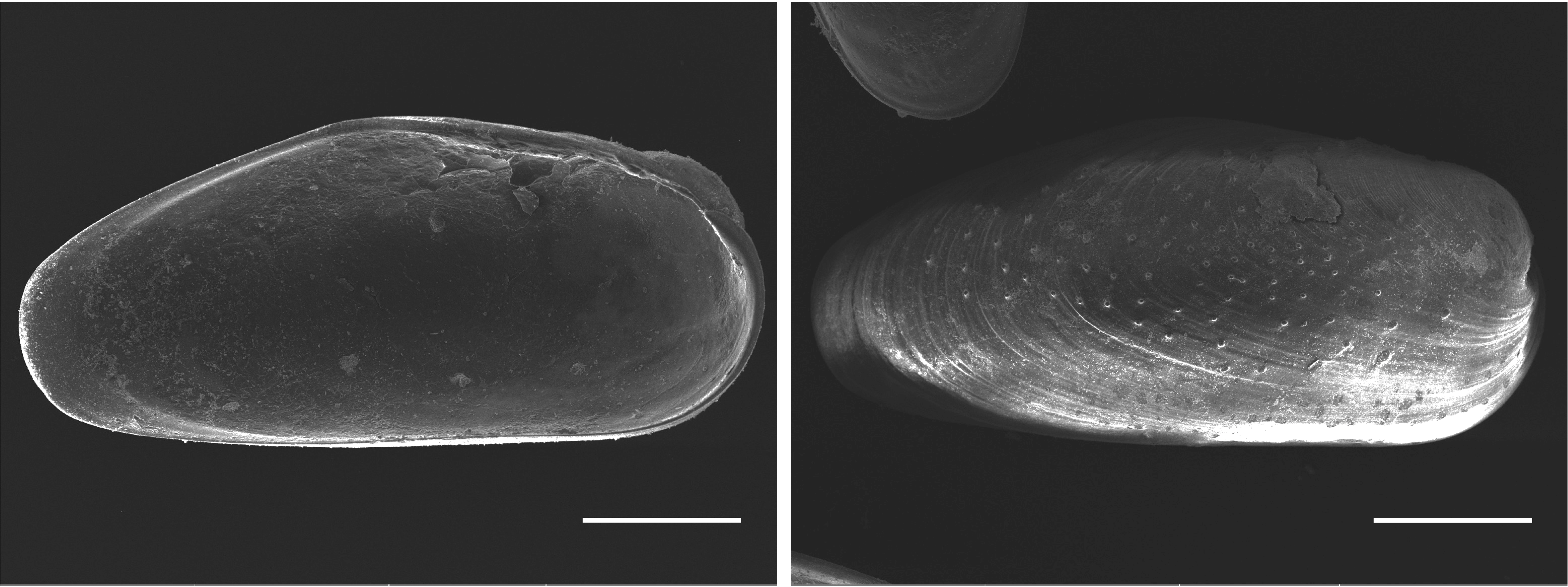

Supplement: S11 Fig — Scale bar 500 μm. (TIFF) [file pone.0202887.s013.tiff]
